# Potato consumption is not associated with cardiometabolic health outcomes in Framingham Offspring Study adults

**DOI:** 10.1017/jns.2022.65

**Published:** 2022-09-02

**Authors:** Ioanna Yiannakou, R. Taylor Pickering, Mengjie Yuan, Martha R. Singer, Lynn L. Moore

**Affiliations:** 1 Preventive Medicine and Epidemiology, Department of Medicine, Boston University School of Medicine, 720 East Concord St, L-518, Boston, MA 02118, USA; 2 Doctoral Program in Biomedical Sciences, Nutrition and Metabolism, Boston University School of Medicine, Boston, MA, USA

**Keywords:** Cardiometabolic risk, Cohort study, Diet, Potatoes, Cup-eq, cup-equivalents, DBP, diastolic blood pressure, FnsV, fruit and non-starchy vegetables, GI, glycaemic index, I, incidence, kcals/d, kilocalories per day, METs, metabolic equivalents, NS, non-starchy, oz-eq, ounce-equivalents, PY, person-years, SBP, systolic blood pressure, T2DM/IFG, type 2 diabetes mellitus or impaired fasting glucose, WHtR, waist-to-height ratio

## Abstract

Some consider potatoes to be unhealthy vegetables that may contribute to adverse cardiometabolic health outcomes. We evaluated the association between potato consumption (including fried and non-fried types) and three key cardiometabolic outcomes among middle-aged and older adults in the Framingham Offspring Study. We included 2523 subjects ≥30 years of age with available dietary data from 3-d food records. Cox-proportional hazards models were used to estimate hazard ratios (HRs) and 95 % confidence intervals (CIs) for hypertension, type 2 diabetes or impaired fasting glucose (T2DM/IFG), and elevated triglycerides, adjusting for anthropometric, demographic and lifestyle factors. In the present study, 36 % of potatoes consumed were baked, 28 % fried, 14 % mashed, 9 % boiled and the rest cooked in other ways. Overall, higher total potato intake (≥4 *v*. <1 cup-equivalents/week) was not associated with risks of T2DM/IFG (HR 0⋅97, 95 % CI 0⋅81, 1⋅15), hypertension (HR 0⋅95; 95 % CI 0⋅80, 1⋅12) or elevated triglycerides (HR 0⋅99, 95 % CI 0⋅86, 1⋅13). Stratified analyses were used to evaluate effect modification by physical activity levels and red meat consumption, and in those analyses, there were no adverse effects of potato intake. However, when combined with higher levels of physical activity, greater consumption of fried potatoes was associated with a 24 % lower risk (95 % CI 0⋅60, 0⋅96) of T2DM/IFG, and in combination with lower red meat consumption, higher fried potato intake was associated with a 26 % lower risk (95 % CI 0⋅56, 0⋅99) of elevated triglycerides. In this prospective cohort, there was no adverse association between fried or non-fried potato consumption and risks of T2DM/IFG, hypertension or elevated triglycerides.

## Introduction

While current dietary advice for Americans encourages the consumption of vegetables as part of a healthy diet^(1)^, potatoes, except sweet potatoes, are considered by some to be ‘unhealthy’ vegetables^(2)^. In the US, potatoes are the most commonly consumed vegetable, accounting for 21 % of all vegetable intake^(3)^. They are a rich and bioavailable source of potassium, dietary fibre and other key nutrients such as magnesium that may benefit cardiometabolic health^(4)^. There is substantial evidence that these nutrients play roles in the prevention of elevated blood pressure^(5,6)^ and other adverse cardiometabolic health outcomes^(7)^. Thus, individuals who regularly consume potatoes as a vegetable or starchy accompaniment to a meal may derive some of these benefits from this food source.

Given the potential contribution of potatoes to overall energy intake, it is important that we study the independent contribution of potatoes to cardiometabolic health. The association between potatoes and various health outcomes may be in part a result of foods that are consumed with them. For example, a prospective study in Spanish adults found that higher potato intake as part of a Mediterranean-style diet was not associated with 4-year changes in blood pressure or hypertension risk^(8)^. Furthermore, a cross-sectional study of an Asian-style diet rich in potatoes (as part of mixed dishes) was associated with a reduced prevalence of diabetes complications^(9)^, while in the large Nurses’ Health Study cohorts, both French fries and total potato consumption were associated with an increased risk of diabetes^(10)^. These disparate findings could be a result of the different ways in which potatoes are cooked and eaten in different population subgroups (e.g. predominantly fried potatoes *v*. baked or boiled or as a part of mixed dishes, including legumes and other vegetables, in other populations). Thus, the observed associations between potato intake and cardiometabolic outcomes in some studies may be explained by or confounded by other factors associated with the overall diet pattern or how these foods are prepared.

In general, few studies have examined the association between potato consumption and cardiometabolic health. A review of 13 observational studies found that the relations between potato intake and risks of obesity, type 2 diabetes mellitus (T2DM) and cardiovascular disease (CVD) were inconsistent^(11)^. Some of these studies found a positive association between French fries and risks of obesity and diabetes. In contrast, a more recent randomised crossover study found that a single serving of potatoes per day as a side dish led to no changes in fasting glucose or other cardiometabolic outcomes^(12)^.

Given the inconsistent evidence and the public health interest in this commonly consumed food, more studies are needed to elucidate the contribution of potatoes to cardiometabolic health. The overall goal of this prospective study is to estimate the effect of potato intake (total, fried and non-fried) among adults ≥30 years of age in the Framingham Offspring Study (diet assessed in 1983–95) on the following three cardiometabolic outcomes during follow-up through 2014: incident type 2 diabetes mellitus or impaired fasting glucose (T2DM/IFG), hypertension and elevated triglyceride levels. We also examined the effects of fried and non-fried potato consumption in combination with other diet and lifestyle factors on the aforementioned cardiometabolic risks.

## Methods

### Study population

For these analyses, study participants were members of the Framingham cohort, which enrolled descendants (and their spouses) of the original Framingham Heart Study, as has been described previously^(13)^. At each Offspring Study examination visit at roughly 4-year intervals, data collection included anthropometric measures, demographic information, haematological samples, blood pressure, medical history and lifestyle habits. Participants were also asked to report any diseases or conditions that had developed since their last visit, including cardiovascular outcomes and diabetes^(14)^. Three-day diet records were collected during the third (1983–7) and fifth exam cycles (1991–5).

**Fig. 1.**
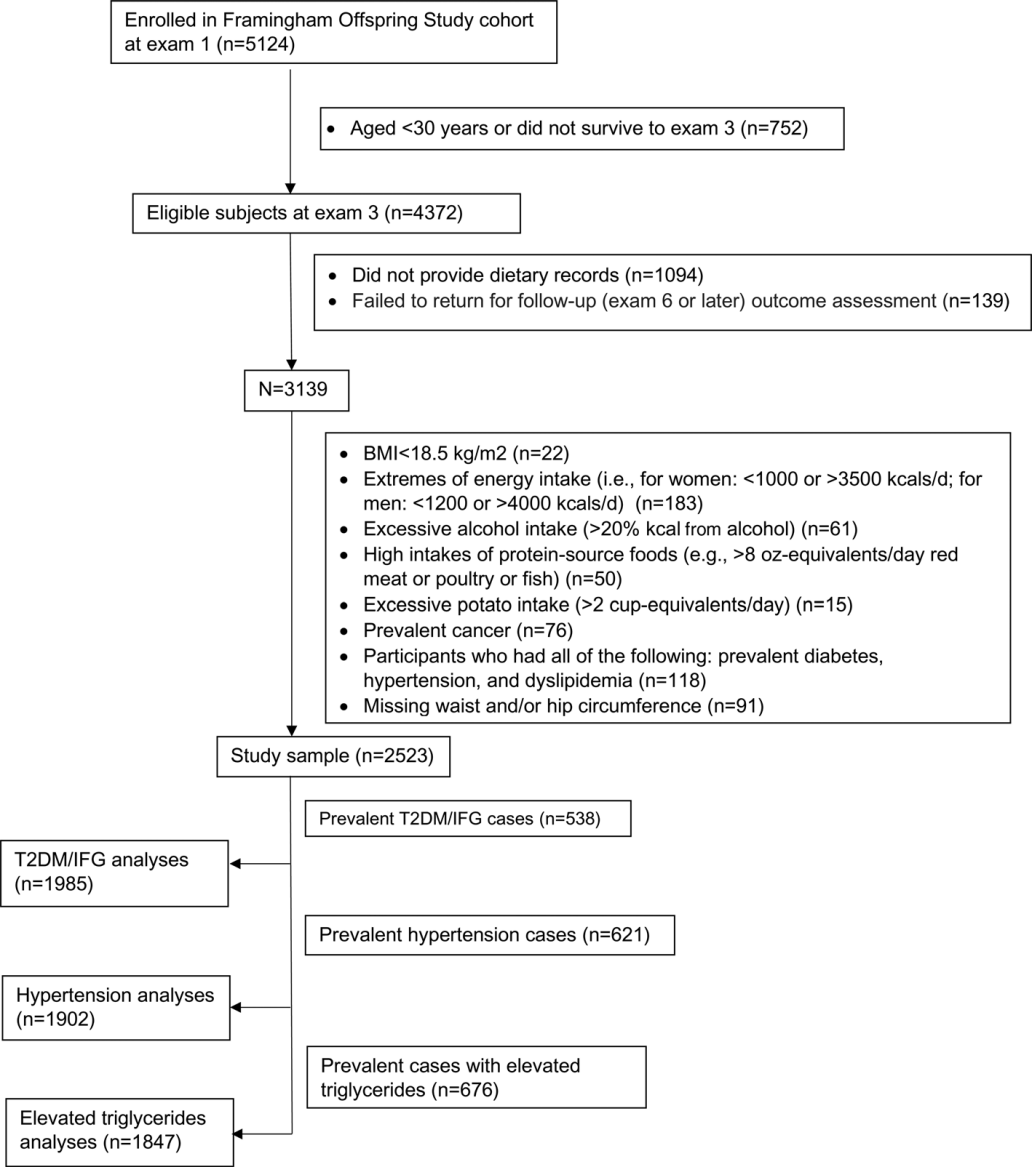
Flowchart of study participants.

The current analyses consisted of 2523 participants who met the inclusion criteria, as shown in Fig. 1. Of the 5124 subjects who were originally enrolled, 3139 returned for examination visit 3 (1983–7), provided at least one set of 3-d dietary records, and had follow-up data for the outcomes of interest to the present study. An additional 22 subjects were excluded with a BMI <18⋅5 kg/m^2^, 309 with excessive intake of diet or alcohol, 76 with prevalent cancer, 118 who had all of the following prevalent disorders – T2DM, hypertension and dyslipidemia, and 91 who were missing waist and/or hip circumferences, leaving a study population of 2523. Finally, for the analyses of each of the three cardiometabolic outcomes, we excluded prevalent cases of that disorder. Specifically, we excluded 538 prevalent cases of T2DM/IFG for those analyses, 621 prevalent cases for the hypertension analyses and 676 with elevated triglyceride levels for the analyses of elevated triglyceride risk. This study was conducted according to the guidelines laid down in the Declaration of Helsinki and all procedures involving human subjects were approved by the Institutional Review Board of Boston University Medical Center (protocol code H-32132). Written informed consent was obtained from all subjects.

### Dietary assessment

Approximately 16 000 d of dietary records were collected from the participants during exams 3 (1983–7) and 5 (1991–5). Nutrient content (including energy, macronutrients, micronutrients and selected phytochemicals) was determined using the Nutrition Data System (NDS) of the University of Minnesota, version 23^(15)^. Linkage was made by the investigators between the NDS food codes and the US Department of Agriculture (USDA) food database, version 06A^(16)^, to calculate each subject's daily intake of foods in each of the USDA food groups^(17)^. The consumption of potatoes (both white and sweet potatoes) was derived from total vegetable servings. In addition, potato consumption was stratified by a cooking method (i.e. fried or not fried) based on food descriptions and ingredients. Fried potatoes included French fries, chips, home-fried and other fried potatoes, while non-fried potatoes included those that were baked, boiled or used as ingredients in non-fried dishes (e.g. soups and other mixed dishes). For these analyses, we estimated each participant's usual potato intake in cup-equivalents (cup-eq) per week as the mean from all days of diet records collected between exams 3 and 5. The serving sizes used for dietary variables were based on the MyPyramid Equivalents Database servings^(17)^.

### Outcome ascertainment

#### Hypertension

At each examination visit, after a short rest in a seated position, blood pressures were measured twice (two minutes apart) by an examining physician using a standard mercury sphygmomanometer and an appropriate-sized cuff. The mean of two measures was used to reflect average SBP and DBP. Hypertension was defined using modified criteria from the seventh report of the Joint National Committee on Prevention, Detection, Evaluation and Treatment of High Blood Pressure^(18)^. A diagnosis of incident hypertension was made when the participant met one of the following criteria: (1) mean SBP of ≥140 mm Hg and/or a mean DBP of ≥90 mm Hg on two separate examination visits, (2) any single visit at which mean SBP was ≥160 mm Hg and/or mean DBP was ≥95 mm Hg or (3) the first visit at which the participant reported taking antihypertensive medication.

#### T2DM/IFG

The diagnosis of incident T2DM was defined as the first exam at which the participant met any of the following criteria: (1) non-fasting glucose of ≥200 mg/dl; (2) fasting (≥10 h) glucose of ≥126 mg/dl; (3) confirmed history of treated diabetes (with oral hypoglycaemic medications or insulin) or (4) a diagnosis of possible diabetes at one exam followed by a diagnosis of definite diabetes at the next exam, without excessive weight gain (≥7 % of body weight) between exams. Subjects who had a fasting glucose level of 100–125 mg/dl but did not meet the above criteria for T2DM were diagnosed with IFG.

#### Elevated triglyceride levels

Fasting triglycerides were measured at each examination visit. Elevated fasting triglycerides were defined as levels ≥150 mg/dl or use of lipid-lowering drugs.

### Potential confounding variables

We explored confounding by a large number of socio-demographic, anthropometric, cardiometabolic and behavioural risk factors. Potential confounding variables were taken from the baseline visit (at the end of the dietary exposure period) and updated at the exam prior to the end of follow-up whenever updated information was available. Potential confounding variables that were explored included age, sex, education level (baseline and updated), anthropometric measures of body fat (i.e. BMI, waist circumference, waist-to-height ratio (WHtR), hip circumference) (at baseline and updated), cigarette smoking (baseline and updated), alcohol intake, total energy intake, daily servings of dairy, fruit and non-starchy vegetables (FnsV), whole grains, total of red meat, poultry and fish, dietary sodium, fibre intakes, total carbohydrate intake, total fat intake, saturated, monounsaturated and polyunsaturated fat intakes, percent of energy from carbohydrates, percent of energy from total fat as well as saturated, monounsaturated and polyunsaturated fats, Healthy Eating Index scores, DASH dietary pattern scores, prevalent T2DM or IFG (except models for incident T2DM or IFG risk), prevalent hypertension (except for models of incident hypertension), prevalent elevated triglyceride levels (except models for triglycerides) and physical activity (baseline and updated).

Self-reported education level was classified as less than college *v*. college or a higher degree. Height and weight were measured using a standard beam balance scale with the subject standing, with shoes off and wearing a hospital gown. The average of all measures of adult height up to age 60 years was used in combination with exam-specific weight measures to calculate exam-specific BMI (kg/m^2^). Mean height was used to reduce random measurement error and the effect of height loss after age 60^(19)^. Waist circumference was measured in a horizontal plane at the level of the umbilicus, while hip circumference was measured at the trochanter major. The WHtR was calculated as the exam-specific waist divided by mean height (in centimetres). A physical activity index was created using self-reported daily hours of moderate and vigorous activity and multiplying each by an appropriate weight based on oxygen consumption required for that level of exercise as follows: sum of (moderate activity hours × moderate activity weight) plus (vigorous activity hours × vigorous activity weight)^(20)^.

### Statistical analysis

Statistical analyses were conducted using SAS software (version 9.4; SAS Institute, Cary, NC). Information on the distribution of potato intake in combination with power considerations was used in sensitivity analyses to select the following cut-off values for four categories of total potato intake: <1, 1 to <2, 2 to <4, ≥4 cup-eq/week. Similarly, three categories of fried and non-fried potatoes (<1, 1 to <2, ≥2 cup-eq/week) were selected. The lowest intake category was used as the reference group for all analyses.

The outcomes of interest included incident T2DM/IFG, hypertension and elevated triglyceride levels. Incidence rates were computed as the number of incident cases occurring in each potato intake category divided by person-years of follow-up in that category as calculated from the end of the baseline dietary assessment to the first of the following events: occurrence of the outcome of interest, loss to follow-up, date of last attended exam (through exam 9, 2011–14) or date of death. Survival analysis was conducted to explore the relations between potato consumption (total, fried, non-fried) and risk of T2DM/IFG, hypertension and elevated triglycerides. Cox-proportional hazards regression models were used to estimate adjusted hazard ratios (HRs) and 95 % confidence intervals (CIs) in each intake category and to test for linear trend across categories.

Confounding was assessed by examining each factor individually in an age- and sex-adjusted model and then in combination with other risk factors (except when factors were colinear). Factors considered to be part of the causal pathway (e.g. fibre intake or potato-derived nutrients) were not included in the models. In a final fully adjusted model, those variables that were found to alter the age- and sex-adjusted HR estimates by 10 % or more were retained. These included age, sex, pack-years of smoking, WHtR, hip circumference, FnsV and red meat intakes. Energy intake and Healthy Eating Index scores did not alter the effect estimates and were dropped from the final models.

Since the impact of fried and non-fried potatoes may differ in combination with other risk factors, we assessed additive effect modification using Cox-proportional hazard models. We used sensitivity analyses and power considerations to dichotomise fried and non-fried potato consumption as well as other potential effect modifying variables. Fried and non-fried potatoes were classified as <2 *v*. ≥2 cup-eq/week. The variables retained as potential effect modifying factors in these analyses included physical activity and red meat. Physical activity (metabolic equivalents per day, METs/d) was dichotomised by combining the lower two quintiles (0–9⋅7 men, 0–8⋅6 women) of activity *v.* the upper three quintiles (9⋅8–54⋅6 men, 8⋅6–49⋅6 women). Red meat intake was categorised as <2 *v*. ≥2 ounce-equivalents/day (oz-eq/d). The proportional hazards assumption in all models was assessed using the weighted Schoenfeld residuals (with the SAS command ZPH). No violations of the assumption were found.

## Results

The median intake of potatoes among these middle-aged and older adults was 2⋅7 cup-eq/week. Among those in the highest potato intake category (≥4 cup-eq/week), the participants consumed 0⋅87 cup-eq/d of potatoes compared with 0⋅04 cup-eq/d in the lowest intake category (Table 1). Furthermore, those in the highest potato intake category consumed 25 % more total fruits and vegetables than those in the lower potato intake category. Thus, potato consumption added to overall fruit and vegetable intake, thereby increasing the likelihood of meeting Dietary Guidelines for vegetables.

**Table 1.** Characteristics of study participants at baseline by total weekly potato consumption in the Framingham Offspring Study

	Total potato intake (cup-equivalents per week)				
	<1	1–<2	2–<4	≥4	
Subjects characteristics	*N* 497	*N* 458	*N* 806	*N* 762	*P*-value*
Gender (% male)	38⋅8 %	36⋅9 %	42⋅4 %	59⋅2 %	*<0*⋅*001*
Age (years)	50⋅3 (0⋅5)	50⋅4 (0⋅5)	50⋅9 (0⋅4)	50⋅3 (0⋅4)	*0*⋅*78*
BMI (kg/m^2^)	26⋅0 (0⋅2)	26⋅7 (0⋅2)	26⋅2 (0⋅2)	26⋅3 (0⋅2)	*0*⋅*56*
Waist-to-height ratio	0⋅524 (0⋅003)	0⋅533 (0⋅003)	0⋅528 (0⋅002)	0⋅533 (0⋅003)	*0*⋅*09*
Alcohol intake (g/d)	10⋅2 (0⋅7)	10⋅0 (0⋅8)	11⋅6 (0⋅6)	11⋅9 (0⋅6)	*0*⋅*02*
Smoking (pack-years)	13⋅7 (0⋅9)	14⋅2 (0⋅9)	16⋅1 (0⋅7)	15⋅6 (0⋅7)	*0*⋅*06*
Physical activity (METs/d)	12⋅7 (0⋅4)	12⋅5 (0⋅4)	12⋅1 (0⋅3)	13⋅1 (0⋅3)	*0*⋅*54*
Education (≥college) (column %)	38⋅8 %	39⋅1 %	30⋅5 %	30⋅6 %	*<0*⋅*001*
Current smoking (column %)	39⋅6 %	40⋅6 %	40⋅0 %	41⋅6 %	*0*⋅*05*
Dietary daily intakes					
Energy intake (kcals)	1775 (19⋅8)	1830 (20⋅6)	1859 (15⋅5)	2045 (16⋅1)	*<0*⋅*001*
Total potatoes (cup-eq)	0⋅04 (0⋅01)	0⋅22 (0⋅01)	0⋅41 (0⋅01)	0⋅87 (0⋅01)	*<0*⋅*001*
Fried potatoes	0⋅01 (0⋅01)	0⋅06 (0⋅01)	0⋅11 (0⋅01)	0⋅26 (0⋅01)	*<0*⋅*001*
Non-fried potatoes	0⋅03 (0⋅01)	0⋅16 (0⋅01)	0⋅31 (0⋅01)	0⋅62 (0⋅01)	*<0*⋅*001*
Total fruit and vegetables (cup-eq)	2⋅80 (0⋅06)	2⋅90 (0⋅07)	2⋅98 (0⋅05)	3⋅48 (0⋅05)	*<0*⋅*001*
Fruit and NS vegetables (cup-eq)	2⋅66 (0⋅06)	2⋅59 (0⋅06)	2⋅47 (0⋅05)	2⋅49 (0⋅05)	*0*⋅*02*
Whole grains (oz-eq)	0⋅70 (0⋅03)	0⋅64 (0⋅03)	0⋅59 (0⋅03)	0⋅58 (0⋅03)	*0*⋅*002*
Meat, poultry, fish (oz-eq)^a^	4⋅51 (0⋅09)	4⋅95 (0⋅09)	5⋅05 (0⋅07)	5⋅59 (0⋅07)	*<0*⋅*001*
Red meat (oz-eq)^b^	1⋅79 (0⋅07)	2⋅17 (0⋅07)	2⋅32 (0⋅05)	2⋅67 (0⋅05)	*<0*⋅*001*
Macronutrients (% of energy)					
Protein	17⋅0 (0⋅2)	17⋅3 (0⋅2)	16⋅9 (0⋅1)	16⋅7 (0⋅1)	*0*⋅*02*
Carbohydrates	47⋅1 (0⋅4)	46⋅7 (0⋅4)	45⋅7 (0⋅3)	45⋅6 (0⋅3)	*<0*⋅*001*
Total fat	34⋅4 (0⋅3)	34⋅5 (0⋅3)	35⋅6 (0⋅2)	35⋅9 (0⋅2)	*<0*⋅*001*
Saturated fat	11⋅8 (0⋅1)	11⋅7 (0⋅1)	12⋅2 (0⋅1)	12⋅2 (0⋅1)	*0*⋅*004*

BMI, body mass index; cup-eq/d, cup-equivalents per day; kcals, kilocalories; METs, metabolic equivalents; NS, non-starchy.

Values are means (standard error) unless otherwise specified. Age was adjusted for sex, all other variables adjusted for sex and baseline age.

a Category includes red meat, processed and organ meats, poultry, fish and other seafood.

b Red meat includes processed and unprocessed red meats.

* *P*-values were generated from ANCOVA for continuous variables and *χ*^2^ tests for categorical variables.

Table 1 shows that age and anthropometric measures of body fat were similar across categories of intake. Compared with the lowest intake category, those with the highest intakes of potatoes were more frequently male and less frequently college graduates. In general, the participants consumed more non-fried than fried potatoes. Furthermore, those who ate more potatoes consumed fewer whole grains and more red meat, poultry and fish. In addition, they had higher energy-adjusted intakes of total and saturated fats but consumed fewer carbohydrates. Supplementary Figure S1 shows the percent of total potatoes consumed that were cooked by different methods in this cohort – 36 % of potatoes were baked, 28 % were fried, 14 % mashed and 9 % boiled. The rest was cooked using other methods, such as soups or mixed dishes (e.g. scalloped potatoes).

Supplementary Table S1 shows the descriptive characteristics of the sample separately for fried and non-fried potato consumption. Here, we observed that those who consumed more fried potatoes (*v*. less) were younger, while the opposite was true for non-fried potatoes. Furthermore, higher fried potato intake was associated with higher fat and saturated fat intakes. It also appears that higher intakes of fried and non-fried potatoes seem to be independent of one another in that those who consumed more fried potatoes tended to consume slightly fewer non-fried potatoes and vice versa.

Tables 2–4 show the adjusted HRs and 95 % CIs for the risk of each cardiometabolic health outcome of interest associated with total potato intake as well as intakes of fried and non-fried potatoes. The incidence rates for T2DM/IFG (Table 2) appeared to increase in a linear fashion across categories of total potato intake, but after adjusting for confounding, there was no association between the amount of total potatoes consumed and the risk of T2DM/IFG. Similarly, there was no association between fried or non-fried potato consumption and the risk of T2DM/IFG. Furthermore, neither total potato consumption nor intake of fried or non-fried potatoes was associated with the risk of hypertension (Table 3) or elevated triglyceride levels (Table 4).

**Table 2.** Hazard ratios for T2DM/IFG associated with intakes of total, fried and non-fried potatoes in the Framingham Offspring Study

	Events/*n*	I/1000 PY	HR^a^	95 % CI
Total potato intakes (cup-eq per week)				
<1	229/398	38⋅0	1⋅00	Ref.
1–<2	220/372	39⋅3	0⋅83	0⋅69, 1⋅00
2–<4	381/637	40⋅4	0⋅87	0⋅73, 1⋅03
≥4	359/578	44⋅4	0⋅97	0⋅81, 1⋅15
*P*-trend				*0⋅96*
Fried potato intakes (cup-eq per week)				
<1	816/1396	39⋅6	1⋅00	Ref.
1–<2	192/291	45⋅8	1⋅10	0⋅94, 1⋅30
≥2	181/298	41⋅6	0⋅95	0⋅80, 1⋅12
*P*-trend				*0⋅87*
Non-fried potato intakes (cup-eq per week)				
<1	377/640	38⋅8	1⋅00	Ref.
1–<2	257/434	39⋅8	0⋅89	0⋅76, 1⋅05
≥2	555/911	42⋅8	0⋅99	0⋅86, 1⋅13
*P*-trend				*0⋅95*

eq, equivalent; I, incidence; PY, person-years; Ref, referent group; T2DM/IFG, type 2 diabetes mellitus or impaired fasting glucose.

a Models were adjusted for sex, age, education, cigarette smoking (pack-years), updated waist-to-height ratio and hip circumference, fruit and non-starchy vegetables and red meat intakes. Models for fried potato intake were also adjusted for non-fried potatoes; those for non-fried potatoes were adjusted for fried potatoes.

**Table 3.** Hazard ratios for hypertension associated with intakes of total, fried and non-fried potatoes in the Framingham Offspring Study

	Events/*N*	I/1000 PY	HR^a^	95 % CI
Total potato intakes (cup-eq per week)				
<1	199/385	31⋅2	1⋅00	Ref.
1–<2	201/361	33⋅3	0⋅91	0⋅74, 1⋅11
2–<4	346/589	34⋅1	0⋅91	0⋅76, 1⋅09
≥4	315/567	33⋅6	0⋅95	0⋅79, 1⋅14
*P*-trend				*0⋅61*
Fried potato intakes (cup-eq per week)				
<1	741/1314	34⋅2	1⋅00	Ref.
1–<2	174/287	34⋅8	1⋅06	0⋅90, 1⋅26
≥2	146/301	27⋅8	0⋅93	0⋅77, 1⋅12
*P*-trend				*0⋅61*
Non-fried potato intakes (cup-eq per week)				
<1	326/632	30⋅2	1⋅00	Ref.
1–<2	236/408	33⋅9	0⋅93	0⋅78, 1⋅10
≥2	499/862	35⋅2	0⋅97	0⋅84, 1⋅12
*P*-trend				*0⋅66*

eq, equivalent; I, incidence; PY, person-years; Ref, referent group.

a Models were adjusted for sex, age, education, cigarette smoking (pack-years), updated waist-to-height ratio and hip circumference, fruit and non-starchy vegetables and red meat intakes. Models for fried potato intake were also adjusted for non-fried potatoes; those for non-fried potatoes were adjusted for fried potatoes.

**Table 4.** Hazard ratios for elevated triglycerides associated with intakes of total, fried and non-fried potatoes in the Framingham Offspring Study

	Events/*N*	I/1000 PY	HR^a^	95 % CI
Total potato intakes (cup-eq per week)				
<1	203/360	36⋅1	1⋅00	Ref.
1–<2	207/345	38⋅6	0⋅94	0⋅77, 1⋅14
2–<4	369/603	40⋅8	1⋅00	0⋅84, 1⋅19
≥4	329/539	40⋅3	1⋅01	0⋅84, 1⋅21
*P*-trend				*0⋅80*
Fried potato intakes (cup-eq per week)				
<1	782/1310	39⋅6	1⋅00	Ref.
1–<2	170/267	42⋅2	1⋅08	0⋅91, 1⋅28
≥2	156/270	35⋅1	0⋅94	0⋅78, 1⋅12
*P*-trend				*0⋅67*
Non-fried potato intakes (cup-eq per week)				
<1	334/584	35⋅9	1⋅00	Ref.
1–<2	240/398	39⋅0	1⋅04	0⋅88, 1⋅23
≥2	534/865	41⋅9	1⋅10	0⋅95, 1⋅27
*P*-trend				*0⋅19*

eq, equivalent; I, incidence; PY, person-years; Ref, referent group.

a Models were adjusted for sex, age, education, cigarette smoking (pack-years), updated waist-to-height ratio and hip circumference, fruit and non-starchy vegetables and red meat intakes. Models for fried potato intake were also adjusted for non-fried potatoes; those for non-fried potatoes were adjusted for fried potatoes.

**Fig. 2.**
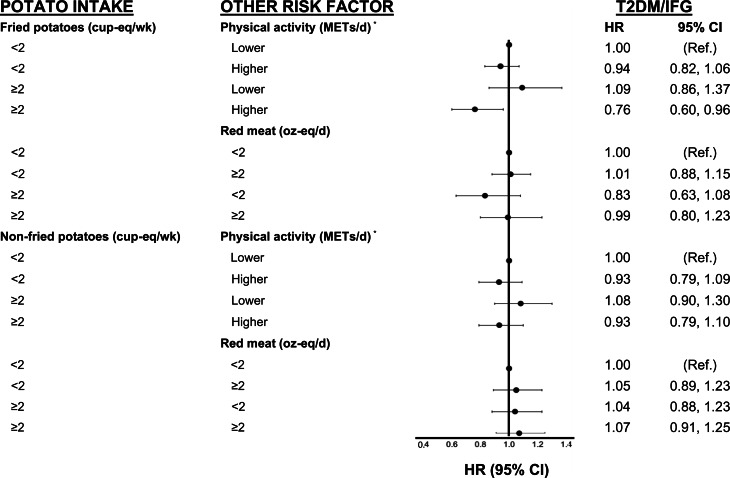
Independent and combined associations of fried/non-fried potato consumption, red meat intake and physical activity with T2DM/IFG in the Framingham Offspring Study. Cox-proportional hazard models for effect modification by physical activity were adjusted for age, sex, education, pack-years of smoking, intake of FnsV and red meat (for physical activity analyses only), and WHtR and hip circumference at the end of follow-up. All models for fried potatoes were also adjusted for non-fried potatoes; those for non-fried potatoes were adjusted for fried potatoes. cup-eq, cup-equivalents; FnsV, fruit and non-starchy vegetables; METs, metabolic equivalents; NS, non-starchy; oz-eq, ounce-equivalents; Ref, referent group; T2DM/IFG, type 2 diabetes mellitus or impaired fasting glucose; WHtR, waist-to-height ratio. *Physical activity was dichotomised based on the lower two quintiles (0–9⋅7 for men, 0–8⋅6 for women) *v*. the upper three quintiles of activity (9⋅8–54⋅6 for men, 8⋅6–49⋅6 for women) (METs/d).

**Fig. 3.**
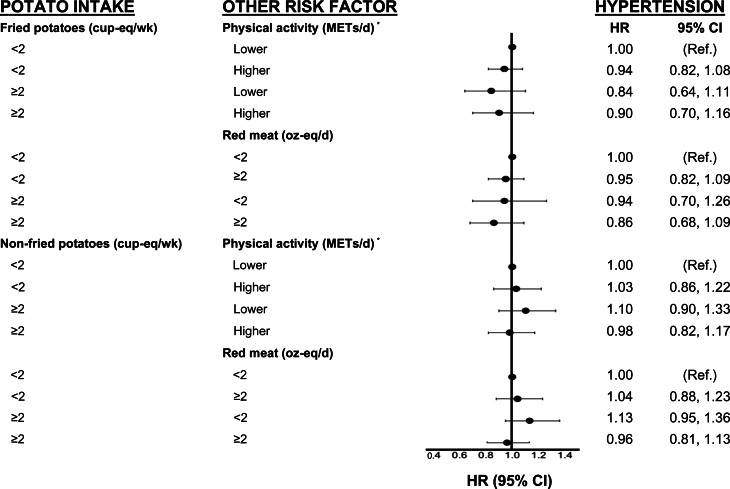
Independent and combined associations of fried/non-fried potato consumption, red meat intake and physical activity with hypertension in the Framingham Offspring Study. Cox-proportional hazard models for effect modification by physical activity were adjusted for age, sex, education, pack-years of smoking, intake of FnsV and red meat (for physical activity analyses only), and WHtR and hip circumference at the end of follow-up. All models for fried potatoes were also adjusted for non-fried potatoes; those for non-fried potatoes were adjusted for fried potatoes. cup-eq, cup-equivalents; FnsV, fruit and non-starchy vegetables; METs, metabolic equivalents; NS, non-starchy; oz-eq, ounce-equivalents; Ref, referent group; WHtR, waist-to-height ratio. *Physical activity was dichotomised based on the lower two quintiles (0–9⋅7 for men, 0–8⋅6 for women) *v*. the upper three quintiles of activity (9⋅8–54⋅6 for men, 8⋅6–49⋅6 for women) (METs/d).

**Fig. 4.**
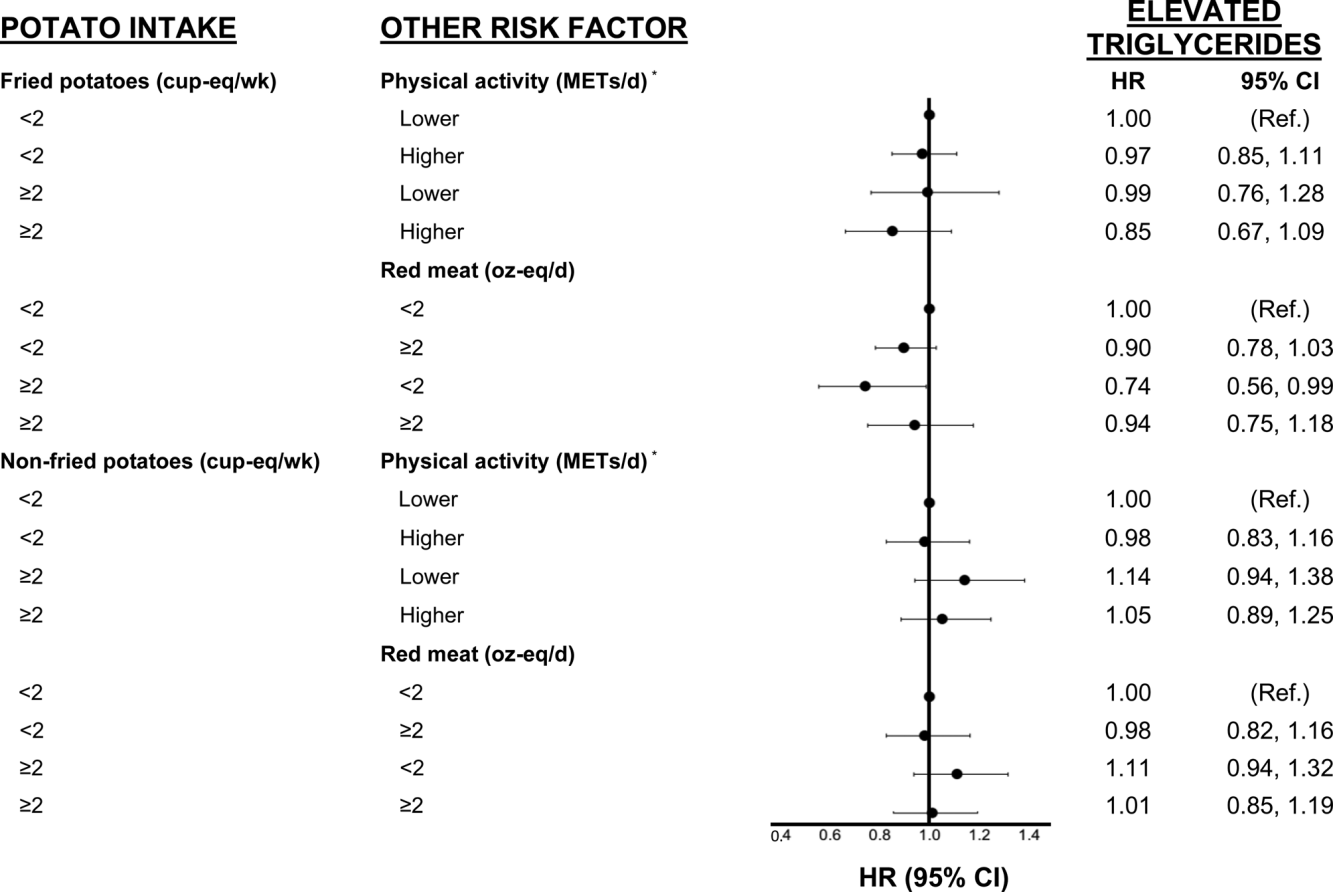
Cox-proportional hazard models for effect modification by physical activity were adjusted for age, sex, education, pack-years of smoking, intake of FnsV and red meat (for physical activity analyses only), and WHtR and hip circumference at the end of follow-up. All models for fried potatoes were also adjusted for non-fried potatoes; those for non-fried potatoes were adjusted for fried potatoes. cup-eq, cup-equivalents; FnsV, fruit and non-starchy vegetables; METs, metabolic equivalents; NS, non-starchy; oz-eq, ounce-equivalents; Ref, referent group; WHtR, waist-to-height ratio. *Physical activity was dichotomised based on the lower two quintiles (0–9⋅7 for men, 0–8⋅6 for women) *v*. the upper three quintiles of activity (9⋅8–54⋅6 for men, 8⋅6–49⋅6 for women) (METs/d).

Figures 2–4 show analyses exploring the independent and combined effects of fried and non-fried potato consumption with two modifying variables. In these analyses, we cross-classified fried and non-fried potato consumption with physical activity and red meat intake. For example, we classified each participant into one of the following four categories: (1) lower potato intake and lower physical activity (referent group), (2) low potato intake and higher physical activity, (3) higher potato intake and lower physical activity and (4) higher potato intake and higher physical activity.

In Fig. 2, we observed that participants who consumed ≥2⋅0 cup-eq/week of fried potatoes and who were more physically active had a 24 % lower risk of T2DM/IFG (HR 0⋅76, 95 % CI 0⋅60, 0⋅96) than less active participants who consumed fewer potatoes. There was no association between non-fried potato intake and the risk of T2DM/IFG. In addition, in Fig. 3, there was no association between either fried or non-fried potatoes in combination with physical activity or red meat consumption and risk of hypertension. Finally, in Fig. 4, we observed that higher fried potato intake among participants with lower red meat intake had a 26 % lower risk (HR 0⋅74; 95 % CI 0⋅56, 0⋅99) of elevated triglycerides compared with those in the referent category. Non-fried potatoes were not associated with elevated triglycerides in these analyses.

## Discussion

In this prospective study of adults with up to 16 years of follow-up, we found no adverse association between potato intake and risk of T2DM/IFG, hypertension or elevated triglycerides. However, participants with higher intakes of fried potatoes who were also more active had a lower risk of T2DM/IFG than other groups. In addition, higher intakes of fried potatoes in combination with lower intakes of red meat were associated with lower risks of elevated triglycerides. Consumption of non-fried potatoes was not associated with any of these cardiometabolic outcomes.

High glycaemic index (GI) foods have been associated with cardiometabolic risk. Since potatoes have a relatively high GI, it is assumed that they will increase cardiometabolic risk as well^(21)^. However, potatoes are typically consumed with foods rich in fat and/or protein which may lower their GI and perhaps explain the absence of an adverse association in this study^(22,23)^. In these analyses, we chose to stratify by red meat to evaluate the common ‘meat and potatoes’ diet. We found no adverse associations of high fried or non-fried (≥2 cup-eq per week) potato intakes combined with higher meat intakes (≥2 ounce-eq per day) on these three cardiometabolic outcomes. Therefore, despite the higher fat content of fried potatoes (compared with other cooking methods), these analyses provide no evidence that fried potatoes have adverse health effects in the outcomes studied in this cohort.

There are very limited human data about the relation between potato intake and dyslipidemia. A previous cross-sectional study in Norway found that overweight/obese men consuming boiled potatoes every day (*v*. once a week) had higher mean triglyceride levels, whereas no such association was found among women^(24)^. Furthermore, a cross-sectional study in Iran found no association between potatoes and lipid levels^(25)^. Results from a clinical crossover trial of healthy adults showed that ingestion of potato fibres lowered postprandial levels of total and esterified cholesterol but had no association with fasting concentrations^(26)^. In the present study, we found that fried potatoes were associated with lower risks of elevated triglycerides among those with lower red meat intakes. These results need to be replicated in future studies.

Potatoes provide a rich source of potassium, dietary fibre and other key nutrients such as magnesium that may be linked with cardiometabolic outcomes, including blood pressure^(27)^. The results of the present study differ from those in the Nurses’ Health Study and Health Professionals Follow-up Study, in which higher intakes of potatoes were associated with a higher long-term risk of developing hypertension^(28)^. In the present study and an earlier study^(29)^, we found no association between total potato intake or intakes of fried and non-fried potatoes and risks of hypertension or pre-hypertension. In contrast with the present results, the analyses from the Nurses’ Health Study and Health Professionals Follow-up Study did not separate potatoes according to cooking methods. Finally, a prospective cohort in two Spanish populations found that neither fried (homemade or commercially prepared) nor baked or boiled potatoes were associated with hypertension risk^(8)^.

There are several mechanisms by which potatoes could benefit cardiometabolic health outcomes. The potassium derived from potatoes is highly bioavailable^(4)^. Since dietary potassium in observational studies of adults has been inversely associated with blood pressure^(5)^, greater flow-mediated dilation^(30)^ and CVD occurrence^(6,31)^, potatoes could provide cardiovascular benefits. However, some studies find no association between potatoes and cardiovascular risk. For example, in analyses from the Swedish Mammography Cohort and a cohort of Swedish men including more than 69 000 subjects, potato consumption was not associated with CVD risk^(22)^. A randomised, crossover trial demonstrated no adverse effects of daily potato consumption (*v*. refined grains) on markers of glycaemia^(12)^. In contrast, other mechanistic studies have found that peptides from potatoes may exhibit angiotensin-converting enzyme inhibitory action, thereby lowering blood pressure^(32)^. In the present study, the fact that apparent beneficial effects were limited to fried potato consumption in certain subgroups indicates that much more research is needed to understand the long-term effects of potato intake and the effects of different cooking methods on cardiometabolic health.

Strengths of the present study include its prospective design in which potato consumption was averaged over 8 years, and subjects were then followed up over the next 16 years for the occurrence of cardiometabolic health outcomes. The prospective design and long-term follow-up reduce the likelihood of reverse causality as an explanation for these results. Furthermore, this relatively large cohort had repeated measures and carefully adjudicated cardiometabolic health outcomes over four sequential exams^(33)^. Moreover, the use of two sets of 3-d food diaries, a ‘gold standard’ method of dietary assessment^(34)^, likely enabled us to derive more accurate estimates of potato intake than some previous studies. Additionally, the averaging of dietary intake over 6 collection days also likely provided more stable estimates of intake^(35)^. Another important strength was the detailed determination of cooking methods, allowing us to accurately separate fried and non-fried potatoes. The careful linkage of foods from the diet records with USDA food groups allowed for a more accurate estimation of potato intake from all sources (as individual foods and as ingredients in composite foods). Finally, the Framingham Offspring Study provided carefully collected data on a wide range of risk factors for the outcomes of interest and enabled us to evaluate change in confounding factors over time, thereby gaining better control for confounding.

There are limitations in the present study as well. As is generally the case in longitudinal studies, dietary intakes were self-reported and, as such, are subject to error (both differential and non-differential) in reporting dietary intake. Physical activity was also self-reported and is therefore subject to error, and, as always, residual confounding cannot be ruled out as an explanation for the results. Furthermore, we were unable to evaluate the effects of sweet and white potatoes separately due to very low intakes of sweet potatoes (only 4⋅5 % of the study population consumed small amounts of sweet potatoes). Another potential concern in this and most long-term studies relates to missing data. Supplementary Table S2 shows the characteristics at the time of enrolment of those who were included and excluded (due to missing data) in the current analyses. Because diet was not assessed at the time of enrolment, these data analyses include a subset of all original members of the cohort who survived and provided data up through the initial follow-up assessment at exam 6. A few differences were noted between the included and excluded participants (e.g. higher smoking rates among the excluded). Since the Framingham Study collected detailed data on a wide range of factors at every exam and since we updated the confounding assessment at follow-up exams, we were able to control for many factors in the analyses that were associated with being excluded. This would reduce the possibility of bias in the observed results. Finally, participants in the present study were exclusively Caucasian, so these results may also not be representative of a multiethnic population.

## Conclusion

Potatoes are an inexpensive source of valuable nutrients. The *2020-2025 Dietary Guidelines* encourage the consumption of vegetables, including potatoes, as part of a healthy diet^(1)^. Still, potatoes (and especially fried potatoes) are believed by many to be less healthy than other vegetables; consequently, some suggest that potato intake should be limited out of concern for cardiometabolic health outcomes^(2)^. The present study adds evidence that moderate consumption of potatoes (whether fried or non-fried) among healthy adults is not associated with an increased risk of T2DM, IFG, hypertension or elevated triglyceride levels.

## Supplementary material

For supplementary material accompanying this paper visit https://doi.org/10.1017/jns.2022.65. click here to view supplementary material

## References

[1] US Department of Agriculture and US Department of Health and Human Services. Dietary Guidelines for Americans, 2020–2025, 9th ed. www.DietaryGuidelines.gov/ (accessed January 2022).

[2] Satija A, Bhupathiraju SN, Spiegelman D, (2017) Healthful and unhealthful plant-based diets and the risk of coronary heart disease in US adults. J Am Coll Cardiol 70, 411–422. 28728684 10.1016/j.jacc.2017.05.047 PMC5555375

[3] US Department of Health and Human Services & US Department of Agriculture (2015) 2015–2020 Dietary Guidelines for Americans, 8th ed. http://health.gov/dietaryguidelines/2015/guidelines/ (accessed January 2022).

[4] MacDonald-Clarke CJ, Martin BR, McCabe LD, (2016) Bioavailability of potassium from potatoes and potassium gluconate: a randomized dose response trial. Am J Clin Nutr 104, 346–353. 27413123 10.3945/ajcn.115.127225

[5] Mente A, O'Donnell MJ, Rangarajan S, (2014) Association of urinary sodium and potassium excretion with blood pressure. N Engl J Med 371, 601–611. 25119606 10.1056/NEJMoa1311989

[6] Pickering RT, Bradlee ML, Singer MR, (2021) Higher intakes of potassium and magnesium, but not lower sodium, reduce cardiovascular risk in the Framingham Offspring Study. Nutrients 13, 269. 33477824 10.3390/nu13010269 PMC7832857

[7] McGill CR, Kurilich AC & Davignon J (2013) The role of potatoes and potato components in cardiometabolic health: a review. Ann Med 45, 467–473. 23855880 10.3109/07853890.2013.813633

[8] Hu EA, Martínez-González MA, Salas-Salvadó J, (2017) Potato consumption does not increase blood pressure or incident hypertension in 2 cohorts of Spanish adults. J Nutr 147, 2272–2281. 29046405 10.3945/jn.117.252254

[9] El Bilbeisi AH, Hosseini S & Djafarian K (2017) Association of dietary patterns with diabetes complications among type 2 diabetes patients in Gaza Strip, Palestine: a cross sectional study. J Health Popul Nutr 36, 37. 29141668 10.1186/s41043-017-0115-z PMC5688727

[10] Halton TL, Willett WC, Liu S, (2006) Potato and French fry consumption and risk of type 2 diabetes in women. Am J Clin Nutr 83, 284–290. 16469985 10.1093/ajcn/83.2.284

[11] Borch D, Juul-Hindsgaul N, Veller M, (2016) Potatoes and risk of obesity, type 2 diabetes, and cardiovascular disease in apparently healthy adults: a systematic review of clinical intervention and observational studies. Am J Clin Nutr 104, 489–498. 27413134 10.3945/ajcn.116.132332

[12] Johnston EA, Petersen KS & Kris-Etherton PM (2020) Daily intake of non-fried potato does not affect markers of glycaemia and is associated with better diet quality compared with refined grains: a randomised, crossover study in healthy adults. Br J Nutr 123, 1032–1042. 31964428 10.1017/S0007114520000252 PMC7282869

[13] Kannel WB, Feinleib M, McNamara PM, (1979) An investigation of coronary heart disease in families. The Framingham Offspring Study. Am J Epidemiol 110, 281–290. 474565 10.1093/oxfordjournals.aje.a112813

[14] Moore LL, Chadid S, Singer MR, (2014) Metabolic health reduces risk of obesity-related cancer in Framingham study adults. Cancer Epidemiol Biomarkers Prev 23, 2057–2065. 25012997 10.1158/1055-9965.EPI-14-0240 PMC4184957

[15] Schakel SF, Sievert YA & Buzzard IM (1988) Sources of data for developing and maintaining a nutrient database. J Am Diet Assoc 88, 1268–1271. 3171020

[16] US Department of Agriculture ARS BHNRC, Food Surveys Research Group. Continuing Survey of Food Intakes by Individuals 1994–96, 1998 and Diet and Health Knowledge Survey 1994–96. USDA Agricultural Research Service, Food Surveys Research Group. Documentation (csfii9498_documentationupdated.pdf). http://www.ars.usda.gov/Services/docs.htm?docid=14521 (Retrieved 05/06/2009).

[17] Bowman SA & Friday JE (2008) MyPyramid Equivalents Database, 2⋅0 for USDA Survey Foods, 2003–2004: Documentation and User Guide. https://www.rs.usda.gov/ARSUserFiles/80400530/pdf/mped/mped2_doc.pdf (accessed January 2022).

[18] Chobanian AV (2003) The Seventh Report of the Joint National Committee on prevention, detection, evaluation, and treatment of high blood pressure. The JNC 7 Report. JAMA 289, 2560. 12748199 10.1001/jama.289.19.2560

[19] Mustafa J, Ellison RC, Singer MR, (2018) Dietary protein and preservation of physical functioning among middle-aged and older adults in the Framingham Offspring Study. Am J Epidemiol 187, 1411–1419. 29590270 10.1093/aje/kwy014 PMC7427817

[20] Kannel WB, Belanger A, D'Agostino R, (1986) Physical activity and physical demand on the job and risk of cardiovascular disease and death: the Framingham study. Am Heart J 112, 820–825. 3766383 10.1016/0002-8703(86)90480-1

[21] Atkinson FS, Foster-Powell K & Brand-Miller JC (2008) International tables of glycemic index and glycemic load values: 2008. Diabetes Care 31, 2281–2283. 18835944 10.2337/dc08-1239 PMC2584181

[22] Larsson SC & Wolk A (2016) Potato consumption and risk of cardiovascular disease: 2 prospective cohort studies. Am J Clin Nutr 104, 1245–1252. 27680993 10.3945/ajcn.116.142422

[23] Hätönen KA, Virtamo J, Eriksson JG, (2011) Protein and fat modify the glycaemic and insulinaemic responses to a mashed potato-based meal. Br J Nutr 106, 248–253. 21338539 10.1017/S0007114511000080

[24] Moholdt T, Devlin BL & Nilsen TIL (2019) Intake of boiled potato in relation to cardiovascular disease risk factors in a large Norwegian cohort: the HUNT study. Nutrients 12, 73. 10.3390/nu12010073 PMC7019529 31892102

[25] Khosravi-Boroujeni H, Mohammadifard N, Sarrafzadegan N, (2012) Potato consumption and cardiovascular disease risk factors among Iranian population. Int J Food Sci Nutr 63, 913–920. 22639829 10.3109/09637486.2012.690024

[26] Cherbut C, Aube AC, Mekki N, (1997) Digestive and metabolic effects of potato and maize fibres in human subjects. Br J Nutr 77, 33–46. 9059228 10.1017/s0007114500002865

[27] Gibson S & Kurilich AC (2013) The nutritional value of potatoes and potato products in the UK diet. Nutr Bull 38, 389–399.

[28] Borgi L, Rimm EB, Willett WC, (2016) Potato intake and incidence of hypertension: results from three prospective US cohort studies. Br Med J 353, i2351. 27189229 10.1136/bmj.i2351 PMC4870381

[29] Yiannakou I, Yuan M, Pickering RT, (2022) Potato consumption is not associated with elevated cardiometabolic risk in adolescent girls. Br J Nutr 128, 521–30. 10.1017/S0007114521003445 34486960

[30] Gijsbers L, Dower JI, Mensink M, (2015) Effects of sodium and potassium supplementation on blood pressure and arterial stiffness: a fully controlled dietary intervention study. J Hum Hypertens 29, 592–598. 25673113 10.1038/jhh.2015.3

[31] Whelton PK (2014) Sodium, potassium, blood pressure, and cardiovascular disease in humans. Curr Hypertens Rep 16, 465. 24924995 10.1007/s11906-014-0465-5

[32] Mäkinen S, Kelloniemi J, Pihlanto A, (2008) Inhibition of Angiotensin converting enzyme I caused by autolysis of potato proteins by enzymatic activities confined to different parts of the potato tuber. J Agric Food Chem 56, 9875–9883. 18841984 10.1021/jf8016817

[33] Feinleib M, Kannel WB, Garrison RJ, (1975) The Framingham Offspring Study. Design and preliminary data. Prev Med 4, 518–525. 1208363 10.1016/0091-7435(75)90037-7

[34] Willett W (2012) Nutritional Epidemiology, 3rd ed. Oxford, New York: Oxford University Press.

[35] Hu FB, Stampfer MJ, Rimm E, (1999) Dietary fat and coronary heart disease: a comparison of approaches for adjusting for total energy intake and modeling repeated dietary measurements. Am J Epidemiol 149, 531–540. 10084242 10.1093/oxfordjournals.aje.a009849

