# Supplementary material for: Potato consumption is not associated with cardiometabolic health outcomes in Framingham Offspring Study adults
Source: J Nutr Sci. 2022 Sep 2;11:e73. doi: 10.1017/jns.2022.65 (PMC9453580; doi:10.1017/jns.2022.65)
Supplement: Supplementary file 1 [file S2048679022000659sup001.docx]

**Supplementary Figure 1.** Percent of total potatoes consumed by different cooking methods. Other potatoes include scalloped potatoes, potato salad, canned potatoes and potatoes in soups and mixed dishes.

| **Supplementary Table 1.** Characteristics of study participants at baseline by fried and non-fried potato consumption in the Framingham Offspring Study^1^. | | | | | | | | | |
| --- | --- | --- | --- | --- | --- | --- | --- | --- | --- |
|  | **Fried potato intake (cup-eq/week)** | | | |  | **Non-fried potato intake (cup-eq/week)** | | | |
|  | **<1** | **1–<2** | **≥2** |  |  | **<1** | **1–<2** | **≥2** |  |
| **Subject characteristics** | **n=1783** | **n=360** | **n=380** | ***P-value^*^*** |  | **n=807** | **n=525** | **n=1191** | ***P-value^*^*** |
| Gender (% male) | 41.1% | 49.4% | 64.2% | *<0.001* |  | 43.9% | 39.8% | 49.7% | *0.005* |
| Age (years) | 51.8 (0.2) | 48.5 (0.5) | 46.1 (0.5) | *<0.001* |  | 48.9 (0.4) | 50.3 (0.4) | 51.7 (0.3) | *<0.001* |
| BMI (kg/m^2^) | 26.2 (0.1) | 26.5 (0.2) | 26.4 (0.2) | *0.25* |  | 26.2 (0.2) | 26.3 (0.2) | 26.3 (0.1) | *0.61* |
| Alcohol intake (gm/day) | 10.9 (0.4) | 11.9 (0.9) | 11.0 (0.8) | *0.69* |  | 10.0 (0.6) | 10.4 (0.7) | 12.1 (0.5) | *0.004* |
| Smoking (pack-years) | 14.5 (0.5) | 15.6 (1.1) | 17.5 (1.1) | *0.01* |  | 14.8 (0.7) | 14.5 (0.9) | 15.6 (0.6) | *0.34* |
| Physical activity (METS/day) | 12.6 (0.2) | 12.5 (0.4) | 12.6 (0.4) | *0.93* |  | 12.5 (0.3) | 12.4 (0.4) | 12.8 (0.2) | *0.39* |
| Education (≥ college) (%) | 33.8% | 33.3% | 33.7% | *0.58* |  | 38.1% | 37.3% | 29.1% | *<0.001* |
| Current Smoking (%) | 40.0% | 42.2% | 41.1% | *<0.001* |  | 39.9% | 41.5% | 40.5% | *0.65* |
| **Energy intake (kcals/day)** | 1845 (11) | 1938 (23) | 2077 (23) | *<0.001* |  | 1835 (16) | 1852 (20) | 1952 (13) | *<0.001* |
| **Food intakes (per 2000 kcals/day)** | |  |  |  |  |  |  |  |  |
| Total potatoes (cup-eq) | 0.39 (0.01) | 0.54 (0.02) | 0.82 (0.02) | *<0.001* |  | 0.18 (0.01) | 0.38 (0.01) | 0.72 (0.01) | *<0.001* |
| Fried potatoes (cup-eq) | 0.02 (0.003) | 0.22 (0.01) | 0.51 (0.01) | *<0.001* |  | 0.14 (0.01) | 0.12 (0.01) | 0.12 (0.01) | *0.07* |
| Non-fried potatoes (cup-eq) | 0.37 (0.01) | 0.31 (0.02) | 0.31 (0.02) | *<0.001* |  | 0.05 (0.01) | 0.25 (0.01) | 0.60 (0.01) | *<0.001* |
| Fruits & vegetables (cup-eq) | 3.49 (0.04) | 3.17 (0.08) | 3.09 (0.08) | *<0.001* |  | 3.16 (0.05) | 3.28 (0.07) | 3.59 (0.04) | *<0.001* |
| Fruit & ns vegetables (cup-eq) | 3.00 (0.04) | 2.52 (0.08) | 2.18 (0.08) | *<0.001* |  | 2.88 (0.05) | 2.81 (0.07) | 2.75 (0.04) | *0.07* |
| Whole grains (oz-eq) | 0.36 (0.01) | 0.29 (0.02) | 0.25 (0.02) | *<0.001* |  | 0.36 (0.01) | 0.33 (0.02) | 0.31 (0.01) | *0.002* |
| Meat, poultry, fish (oz-eq) † | 2.78 (0.03) | 2.75 (0.06) | 2.70 (0.06) | *0.17* |  | 2.58 (0.04) | 2.77 (0.05) | 2.89 (0.03) | *<0.001* |
| Red meat (oz-eq) ‡ | 1.18 (0.02) | 1.29 (0.04) | 1.29 (0.04) | *0.002* |  | 1.09 (0.03) | 1.17 (0.03) | 1.31 (0.02) | *<0.001* |
| **Macronutrients (% of energy)** |  |  |  |  |  |  |  |  |  |
| Protein | 17.2 (0.1) | 16.5 (0.2) | 15.9 (0.2) | *<0.001* |  | 16.6 (0.1) | 17.0 (0.1) | 17.1 (0.1) | *0.001* |
| Carbohydrates | 46.8 (0.2) | 45.0 (0.4) | 44.1 (0.4) | *<0.001* |  | 46.6 (0.3) | 45.8 (0.3) | 45.9 (0.2) | *0.08* |
| Total fat | 34.4 (0.2) | 36.4 (0.3) | 38.2 (0.3) | *<0.001* |  | 35.3 (0.2) | 35.4 (0.3) | 35.1 (0.2) | *0.50* |
| Saturated fat | 11.8 (0.1) | 12.3 (0.2) | 12.8 (0.2) | *<0.001* |  | 12.1 (0.1) | 12.0 (0.1) | 12.0 (0.1) | *0.69* |
| eq, equivalent; BMI, body mass index; gm, grams; METs, metabolic equivalents; mg, milligrams; ns vegetables, non-starchy vegetables, oz-eq, ounce equivalents.  Values are means (standard error) unless otherwise specified. Age was adjusted for sex, all other variables adjusted for sex and baseline age.  *P-values were derived from analysis of variance models for continuous variables and chi-square for categorical variables. | | | | | | | | | |
| †Category includes red meat, processed and organ meats, poultry, fish and other seafood. ‡Red meat includes processed and unprocessed red meats. | | | | | | | | | |

| **Supplementary Table 2.** Characteristics of study participants at exam 1 among participants who were included and excluded from the analysis data set in the Framingham Offspring Study. | | | |
| --- | --- | --- | --- |
|  | **Included** | **Excluded** |  |
| **Subject Characteristics** | **N=2523** | **N=2601** | ***P value**** |
| Age (years) | 36.5 (10.0) | 37.0 (10.9) | *0.13* |
| Height (inches) | 65.9 (3.7) | 65.9 (3.9) | *0.70* |
| BMI (kg/m^2^) | 24.8 (4.2) | 25.6 (4.8) | *<.0001* |
| Systolic blood pressure (mm Hg) | 120.7 (14.8) | 123.1 (17.9) | *<.0001* |
| Diastolic blood pressure (mm Hg) | 78.1 (10.0) | 79.1 (11.7) | *0.0008* |
| Alcohol intake (gm/day) | 13.0 (0.3) | 16.7 (0.5) | *<.0001* |
| Smoking (pack-years) | 10.2 (0.3) | 15.2 (0.4) | *<.0001* |
| Physical activity (METs/day) † | 9.4 (8.9) | 9.7 (8.9) | *0.28* |
| Education (≥ college) (column %) | 33.7% | 22.3% | *<0.001* |
| Current smoking (column %) | 36.7% | 52.0% | *<0.001* |
| Sex (column % female) | 54.2% | 48.9% | *0.0002* |
| BMI, body mass index and METs, metabolic equivalents.  Values are means (standard deviation) unless otherwise specified. Age was adjusted for sex, all other variables adjusted for sex and baseline age.  **P values* were generated from two sample t-test for continuous variables and chi-square tests for categorical variables.  † Due to unavailability of these data at exam 1, physical activity was determined at exam 2 (retained n=2241; excluded n=1618). | | | |
|  | | | |
|  | | | |
